# The Influence of Heart Rate on Peripheral Vascular Function Among Pacemaker Patients

**DOI:** 10.7150/ijms.103341

**Published:** 2025-01-01

**Authors:** Md Rizman Md Lazin Md Lazim, Kalaivani Chellappan, Azizah Ugusman, W Yus Haniff W Isa, Mohd Shawal Faizal Mohamad, Wan Amir Nizam Wan Ahmad, Amilia Aminuddin

**Affiliations:** 1 Department of Physiology, School of Medical Sciences, Health Campus, Universiti Sains Malaysia, Kubang Kerian, 16150 Kota Bharu, Kelantan, MALAYSIA.; 2 Department of Physiology, Faculty of Medicine, Universiti Kebangsaan Malaysia, Jalan Yaacob Latif, Bandar Tun Razak, 56000 Cheras, Kuala Lumpur, MALAYSIA.; 3 Department of Electrical, Electronic and Systems Engineering, Faculty of Engineering & Built Environment, Universiti Kebangsaan Malaysia, 43600 Bangi, Selangor, MALAYSIA.; 4 Department of Internal Medicine, School of Medical Sciences, Health Campus, Universiti Sains Malaysia, Kubang Kerian, 16150 Kota Bharu, Kelantan, MALAYSIA.; 5 Department of Internal Medicine (Cardiology Unit), Hospital Canselor Tuanku Muhriz, Jalan Yaacob Latif, Bandar Tun Razak, 56000 Cheras, Kuala Lumpur, MALAYSIA.; 6 Biomedicine Programme, School of Health Sciences, Health Campus, Universiti Sains Malaysia, Kubang Kerian, 16150 Kota Bharu, Kelantan, MALAYSIA.

**Keywords:** cardiovascular, HR, photoplethysmography, fitness index, vascular age, pacemaker

## Abstract

**Background:** The finger photoplethysmography fitness index (PPGF), a marker of peripheral vascular function, has been linked to heart rate (HR) variability. However, the influence of acute HR changes on resting PPGF, a purported indicator of local blood flow, remains unclear.

**Objective:** This study aimed to determine the influence of acute HR changes on resting PPGF.

**Methods:** A total of 22 pacemaker recipients (mean age: 52.27 ± 10.43 years) underwent a controlled study. Baseline assessments included demographics, blood pressure (BP), blood analysis, PPGF, and vascular functions. HR was progressively increased from 70 bpm to 90 bpm in 10 bpm increments with 20 min resting periods at baseline and between pacing intervals. HR, PPGF, and BP were recorded at each pacing level.

**Results:** Systolic and diastolic BP increased with rising HR. Conversely, PPGF remained stable across different HR levels (70 bpm: 51.02 ± 11.52%, 80 bpm: 51.15 ± 11.82%, 90 bpm: 49.73 ± 11.55%; p > 0.05), suggesting that resting PPGF is independent of acute HR fluctuations.

**Conclusion:** Our findings demonstrate that PPGF accurately reflects local blood flow, unaffected by short-term HR variations. This study supports the use of PPGF as a reliable marker for vascular health and age assessment, even in individuals with fluctuating HR, such as older adults with multiple comorbidities. Further research is warranted to establish the applicability of PPGF in younger, healthier populations.

## Introduction

Cardiovascular diseases (CVDs) are the major cause of deaths worldwide 1. About 17.9 million people died due to CVD in 2019, accounting for 32% of world deaths 1. In particular, 85% of these deaths were due to heart attack and stroke 1. More than three quarters of deaths due to CVD happened in low- and middle-income countries 1. In 2022, the major causes of death in Malaysia were 16.1% due to ischemic heart disease (IHD), 13.3% due to pneumonia, and 7.2% due to cerebrovascular diseases (Department of Statistics Malaysia, 2023). IHD is the major cause of death among individuals aged 41-59 years (20%) and 60 years and above (16.7%) and among males (18.2%) 2. IHD was also the major cause of death among Malay (17.3%), Chinese (14.7%), and Indian (21.1%). The circulatory system was the primary cause of death in Ministry of Health hospitals in 2022 (20.79%) and in private hospitals in 2022 (23.09%) 3. The increase in CVD prevalence is largely due to an increase in the prevalence of CVD risk factors, such as smoking, overweight, dyslipidemia, hypertension, and diabetes 4. Awareness among the population, screening activities by health providers, and early diagnosis are important for managing these risk factors in ensuring that CVD prevention can be taken during an earlier stage. The mortality rate due to CVD was proven to decrease by 50% in high-income countries, such as Australia, Canada, and the United States, from 1965 to 1990 by employing this strategy 5.

Several recommendations have been made for screening CVD risk factors, such as hypertension and dyslipidemia, starting from young adults 6-8. However, screening practice among the young population is low and may contribute to the increment of CVD risk factors 9. The low screening rate may be due to low awareness level, high screening cost, and lack of time. An improvement in the screening process that includes a simple test and shorter duration may enhance the effectiveness of screening activities among this population. The incorporation of a noninvasive marker that accurately reflects vascular health exhibits the potential to promote and encourage a habit of frequent screening among individuals.

**Figure 1:**
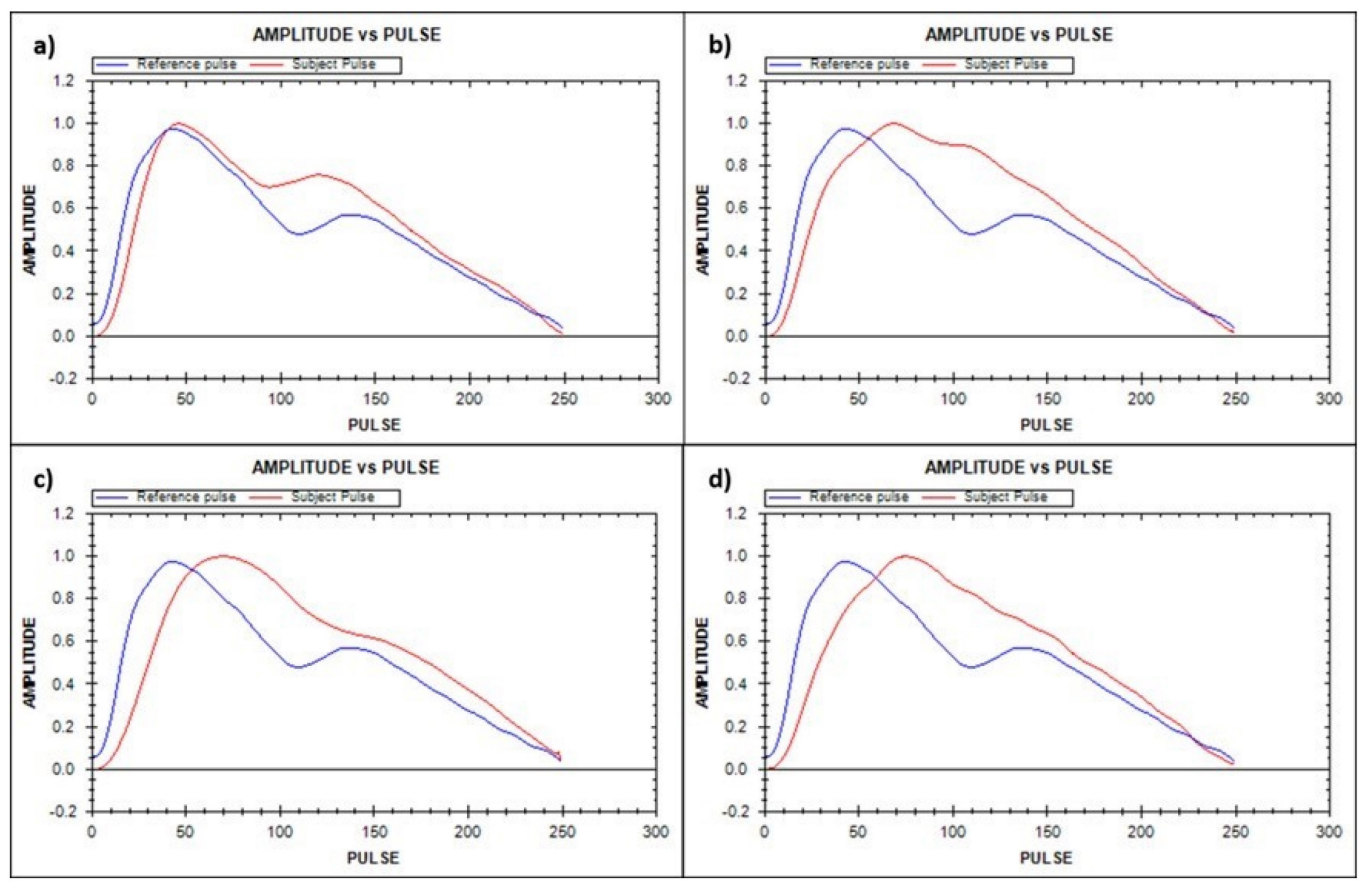
PPG single-pulse plot for different CVD risk factors: (a) healthy, (b) diabetes, (c) hypercholesterolemia, and (d) hypertension. Blue line = reference pulse; red line = subject pulse 14.

Apart from the conventional risk factors mentioned above, several cardiovascular risk markers, such as pulse wave velocity (PWV), aortic pressure (AP), or augmentation index (AI), have been introduced as markers of vascular function, while C-reactive protein (CRP), has been introduced as a marker of inflammation 10. These methods are expensive, and some of them are invasive, limiting their acceptance among the population. The development of the finger photoplethysmography (PPG) fitness index (PPGF) as a marker of vascular function may be a revolutionary solution. Finger PPG is a noninvasive technique that measures blood volume changes of the finger arterial bed by transmitting or reflecting light source 11. PPGF was developed by Chellappan and Mohd Ali 12 as an early screening marker to identify individuals who are developing CVD risk factors. PPGF was formulated from an alternating current (AC) component of PPG morphology change compared with a 19-year-old healthy Malaysian subject 12. The AC component of PPG records the arteries' physiological properties 13. The PPGF algorithm was developed with reference to age and differences between healthy subjects and subjects with CVD risk factors (hypertension, diabetes mellitus, dyslipidemia, obesity, and smoking). A higher PPGF value reflects better function of the blood vessels. Figure 1 illustrates the PPG single-pulse plot of subjects with different CVD risk factors: (a) healthy, (b) diabetes, (c) hypercholesterolemia, and (d) hypertension 14. The risk factors of the subjects are verified through biochemical assessment. A previous study found that PPGF was reproducible, and the sensitivity of this technique was about 80% among the young population 15, 16.

Vascular age is an aging index for the vascular system 12. A linear association exists between the ages documented in 72 healthy subjects and PPGF 12. The best fit of the line slope tends to decrease with age addition. Therefore, the aging index known as vascular age is based on the best fit of the line slope for a given subject 12. The alterations in the whole PPG pulse contour comparison for age-related alterations have revealed a constant and progressive alteration due to aging 12.

Previous studies have shown that variation in heart rate (HR) affects vascular function measurement, such as AI, which serves as an indirect measure of arterial stiffness 17, 18, and PWV, which is a direct measure of aortic stiffness 19-21. PPGF has also been found to be affected by HR 22. HR exerted a significant negative effect on PPGF at a lower blood pressure (BP), and this effect was reduced gradually when BP increased 22. PPGF reduction due to HR may be facilitated by aortic compliance reduction with alterations in BP 22. However, PPGF reflects local blood volume changes because it is measured at the fingers and is primarily influenced by local metabolites and myogenic activities and not by central factors 23-25.

Thus, any change in central circulation, such as increased BP or HR, may not affect PPGF under resting conditions. Our previous study also showed that the effects of HR were statistically insignificant 14. We speculated that the previous methodology of continuous pacing without appropriate resting might stress patients and their circulation, increasing the sympathetic activity that resulted in changes in PPGF. We modulate the methodology by adding a rest period before each pacing activity in the present study. Identifying the effects of HR variation on PPGF is important because its validity may be disputed among subjects with day-to-day HR variation.

## Materials and Methods

In this study, an investigation on peripheral vessel characteristic changes due to HR variation among subjects at rest was conducted. CVD patients with a pacemaker were selected to allow HR variation engagement. A similar study was conducted by Aminuddin *et al.* (2018) on 20 patients with pacemaker. A minor modification in the protocol was implemented in the current study compared with that in the previous study 22. The present study implemented a resting period of 20 min at baseline HR after each pacing. The previous study was conducted without a resting period between each selected pacing 22. A resting period of 20 min was chosen by referring to a previous study that recorded a 20 min duration as the threshold for PPG waveform recovery after exercise 26. The designed study protocol that involved humans was in accordance with the guidelines of the Declaration of Helsinki, and the protocols were reviewed and approved by the Universiti Kebangsaan Malaysia (UKM) Research Ethics Secretariat and the Universiti Sains Malaysia (USM) Human Research Ethics Committee (HREC).

### Recruitment of Subjects

This study was approved by the UKM Research Ethics Secretariat (reference no.: UKM PPI/111/8/JEP-2018-328) and USM HREC (study protocol code: USM/JEPeM/19060360). The subjects were selected from Hospital Canselor Tuanku Muhriz (HCTM) and Hospital USM (HUSM) in their echocardiogram clinics. All the recruited patients were permanent pacemaker users. These patients were from a regular follow-up cohort without any hospitalization record. Informed consents were obtained from all the subjects.

### Sample Size Calculation

For this study, the minimum number of subjects that should be recruited was 15. The number was calculated to identify a 2% change in PPGF, with a standard deviation of 5.66%, an alpha level of 5% and a power of 80% 27. A total of 22 patients met the inclusion criteria and were included in this study.

### Subject Selection Criteria

The subjects were patients with pacemaker who had been screened for regular follow-up in the echocardiogram clinic. The selected patients were within the range age of 18-70 years old, with a paced rhythm of <70 bpm. The exclusion criteria were carefully designed to ensure the safety of the patient in performing the procedure. Patients who experienced myocardial infarction within last 12 months were excluded due to the expectation that they might develop angina or heart attack during pacing. The medical team went further to exclude patient with implantable cardioverter defibrillators considering the complication that might arise when varying pacing due to the synchronization between two devices.

This study excluded patients with potential unstable or stable angina, such as uncontrolled hypertension, i.e., systolic BP (SBP)/diastolic BP (DBP) of >160/100 mmHg, left ventricular ejection fraction (LVEF) of <40%, congestive heart failure, or hemoglobin A1c (HbA1c) of >6%. Among patients with angina, manually induced HR variations can precipitate a range of adverse outcomes. For example, increased HR during an angina attack may exacerbate symptoms and potentially provoke a myocardial infarction. Conversely, decreased HR can reduce coronary blood flow, leading to chest pain or discomfort. A rigorous exclusion criterion was implemented to account for all potential risk factors associated with HR variability among patients with pacemaker. The inclusion and exclusion criteria are listed in Table 1.

### BP, Body Weight and Height, and Measurement of Blood Parameters

Participating subjects were required to fast for at least 4 h prior to all measurements, including PPGF and PWV. SBP and DBP were measured (Welch Allyn Propa® CS 244, USA) after 5 min of resting. Body weight and height were measured using a wall-mounted stadiometer (SECA, Germany) and a weighing scale (SECA, Germany). The blood samples of the subjects were withdrawn from the antecubital veins and sent to Gribbles Laboratory for further analysis of full blood count, lipid profiles, glucose level, and uric acid level.

### PPGF Measurement and Vascular Age Assessment

PPGF measurements were taken while the participants were in a supine position in a temperature-controlled environment set between 20 °C and 25 °C. The participants rested for 20 min before a finger probe (OEM-601, Dolphin Medical, Inc.) was attached to their right index finger, with data recorded for a period of 2 min. PPGF was derived by analyzing morphological variations in the AC component of the PPG signal and compared against a gender-specific reference obtained from a healthy 19-year-old individual within the population and patients with cardiovascular modifiable, unmodifiable, and intermediate risk factors from the formula below:



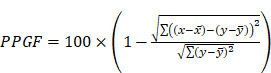



where *x* is the reference pulse amplitude, and *y* is the tested pulse amplitude 15. In this study, data were recorded using NiVARix 1.0, a UKM in-house developed and validated device embedded with PPGF and vascular age computation 14,15. Vascular age was calculated using a predictive model developed in 2009; it was based on a sample of 300 subjects aged 19-70 years, with an equal distribution of genders 14.

### PWV, AP, and AI Measurement

Subsequently, AP, AI, and PWV were measured using an arteriograph (TensioMed Ltd., Budapest, Hungary). Measurements involved placing a cuff around the right upper arm of the subject, and then the cuff was inflated at supra-systolic pressure. AI (%) was calculated using the following formula 28:

AI (%) = [(P2-P1)]/PP×100

where P1 is the amplitude of the first wave, while P2 is the amplitude of the late systolic wave. PP is the pulse pressure. The measurement of PWV involves the device that estimates the time between the first and late systolic waves, i.e., return time (RT). PWV was calculated as the distance measured from the sternal notch to the symphysis pubis (Jug-Sy) (in meters) divided by RT/2 (in seconds) 28.

PWV (m/s) = (Jug-Sy)/([RT/2])

**Figure 2:**
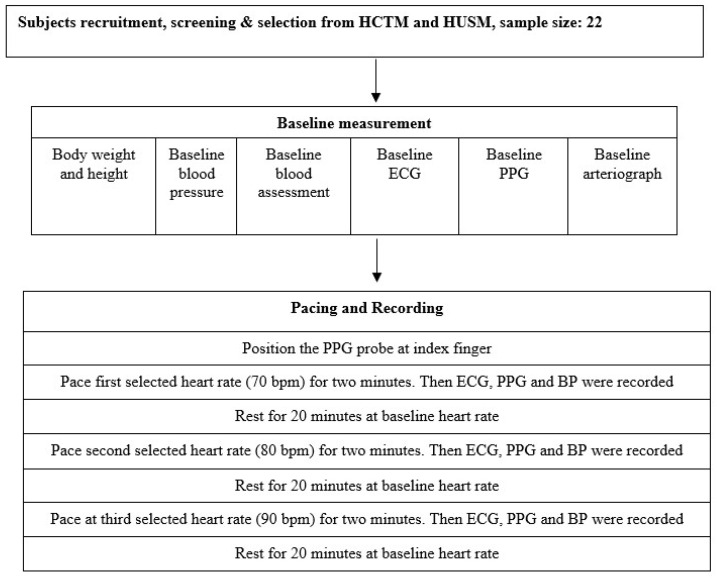
Flowchart of parameter measurement. HCTM, Hospital Canselor Tuanku Muhriz; HUSM, Hospital Universiti Sains Malaysia; ECG, electrocardiogram; PPG, finger photoplethysmography; BP, blood pressure.

The calculation of the aortic SBP is based on the relationship between the brachial and aortic SBP 28. Upon completing the above recording, the patients rested for 10 min in a supine position while preparing for the pacing procedure. Electrocardiogram (ECG) was used to monitor HR and rhythm. The summary of all the data acquisition procedures, from the subjects' recruitment for the pacing and recording procedure, is illustrated in Figure 2. PWV and AI were only measured at baseline and not during pacing due to technical difficulty and patient safety consideration.

### Safety and Precaution

Strict adherence to safety protocols was prioritized during data collection to safeguard patient well-being. All the participants were confirmed to be free from heart failure by a cardiologist prior to inclusion. The study environment and equipment were selected in accordance with Department of Occupational Safety and Health 29. Patient enrollment was based on recommended safe HR parameters 30. Continuous cardiac monitoring by a cardiologist and ECG recording by a medical officer were implemented throughout the data collection process.

### Data Analysis

Data were analyzed using SPSS software version 24. The difference between vascular and chronological ages was determined via a paired T-test. The changes in PPGF, BP, HR, and vascular age as HR increased were analyzed via generalized linear model repeated measures. A *p*-value less than 0.05 was considered significant.

## Results

Table 2 provides the subjects' baseline characteristics. The mean age of the subjects (chronological age) was 52.27±10.43 years old. The mean body mass index (BMI) was 25.96±3.77 kg/m^2^, within the overweight range. The mean CRP was 3.48±3.37 mmol/L, higher than the normal range, indicating an increased risk for CVD 31. The mean baseline AI and PWV were 41.31±15.32% and 9.46±1.99 m/s respectively, which were higher than the normal range 32, 33. The most prevalent CVD risk factors were overweight and obesity (72.7%), followed by dyslipidemia (68.2%). The chronological age was 29-64 years and vascular age was 37-94 years. No significant difference was found between vascular age and chronological age (*p* = 0.26).

In particular, 21 participants had dual-chamber pacemakers, while 1 had a ventricular sensing and pacing device (Table 3). No subjects exhibited heart failure. The primary indications for pacemaker implantation were sick sinus syndrome and heart block. SBP, DBP, and mean arterial pressure (MAP) increased significantly with rising HR. Conversely, PPGF and vascular age remained stable across HR increments (Table 4).

## Discussion

This study is important because it determines the effect of PPGF with changes in HR on the same subject. If no difference in PPGF occurs with changes in HR, then PPGF is reliable for use as an early screening and monitoring tool for communities in the future. In addition, the ability to identify blood volume alteration in the peripheral blood vessels of the finger makes PPG an uncomplicated, affordable, and safe optical method. This study discovered that PPGF is not altered as HR increases due to the physiological fact that local blood flow is unaffected by HR changes, as reflected in PPG measurement. Local blood flow is mostly regulated by local autoregulation, which is determined by the presence of local metabolites 23-25, 34 and the myogenic activity of the arteriole 23-25, 34, 35.

In the local autoregulation mechanism, local metabolites, such as carbon dioxide, lactic acid, potassium ions, and adenosine, will accumulate with an increase in tissue activity. Local oxygen concentration will also be reduced. The reduction in O_2_ tension and pH leads to precapillary sphincter relaxation and increased blood flow. The local reduction of O_2_ also stimulates a vasodilatory gene and produce hypoxia-inducible factor-1α (HIF-1α) 34, 36-39. HIF-1α regulates genes that increase oxygen delivery and facilitate metabolic adaptation to hypoxia 40. By contrast, when metabolites are less, blood flow to the tissue will be reduced via arteriolar vasoconstriction. This regulation assists in controlling blood flow in accordance with the need of the tissue. Blood flow should remain constant at rest due to less activities and no accumulation of metabolites.

Simultaneously, studies have observed an increase in SBP due to increased HR 24, 41-45. Increased HR leads to an increase in cardiac output (CO), leading to an elevation of SBP 24, 41-45. Increasing BP initially leads to increased blood flow, which increases the stretching of arterioles 34, 35, 46, 47. This phenomenon stimulates the arteriole myogenic activity mechanism, which leads to vasoconstriction, also known as pressure-stimulated vasoconstriction. In detail, the increased stretching of blood vessel leads to the opening of mechanically gated cation channels that causes small depolarization and stimulates the opening of more surface-membrane voltage-gated Ca^2+^ channels 24, 46-48. The subsequent entering of Ca^2+^ stimulates smooth muscle contraction, enhancing myogenic vessel tone and leading to vasoconstriction, which causes increased resistance 24, 34, 35, 46, 47. Vasoconstriction reduces blood flow and makes it return to normal. In addition, increased local blood flow due to increased MAP leads to increased O_2_ levels, and metabolites are flushed away more rapidly 23, 24, 34, 44. These local chemical alterations lead to local arteriolar vasoconstriction, resulting in compensatory blood flow reduction 23, 24, 44. Thus, local blood flow is fairly maintained despite variation in BP via the two aforementioned mechanisms. No increase in PPGF is also evidenced with an acute increase in SBP, proving the fact that PPGF measurement represents local blood flow changes.

In the current study, PPGF measurements were obtained under resting conditions to maintain a stable blood flow, potentially explaining the unchanged PPGF despite increased HR. However, several alternative explanations for the consistent PPGF values warrant consideration. First, the modest HR increments may have been insufficient to induce PPGF alterations. Nevertheless, the investigated HR range falls within normal limits and is suitable for accommodating daily resting HR fluctuations. HRs that exceed 100 beats per minute are typically considered abnormal and beyond the resting state. Second, the influence of various pharmacological treatments received by the patients could have confounded the results. Given the chronic conditions prevalent in pacemaker recipients, this limitation is inherent in studies that involve this population 17, 22. Third, a preexisting alteration in baseline PPGF might have reduced its responsiveness to BP changes. This possibility is unlikely given the implementation of a 20 min resting period prior to baseline measurement and the 20 min rest intervals between HR pacing sessions. Lastly, the timing of HR elevation may have been suboptimal. However, the 2 min duration of increased HR employed in this study, compared with 1 min in our previous research 22, appears adequate, as evidenced by the concomitant rise in BP.

This finding contrasted with our previous study conducted among patients with pacemaker 22. In the previous protocol, HR was paced without a resting period among selected paced HRs. We contemplated that the decreased PPGF in the previous study was due to an increased in sympathetic nerve activity (SNA), which is also known to cause peripheral arterial stiffness (AS) 49, 50 and may affect PPG 51. Increased SNA may occur due to increased myocardial oxygen demand. The continuous pacing of the heart leads to an increase in its workload and a myocardial need for oxygen 52, reducing diastolic time, and consequently, coronary artery perfusion 53. Although our previous study was only paced at 1 min, an additional paced of HR, which was at 60 bpm and 100 bpm, was provided. Without appropriate resting between paced HR, this intervention may be predisposed to the accumulation of metabolites, such as adenosine and CO_2_, and decreased oxygen and pH, which stimulates the peripheral chemoreceptor and leads to the activation of SNA 54. Increased SNA may also occur in subjects who are anxious to know that their HR will be modulated. This effect will be less substantial by adding resting stations because the resting period contributes to the subject's psychological stability. Third, sympathetic activity can also be potentially increased due to increased oxidative stress as BP increases 55-57. However, TPR did not increase as expected in the previous study 22, given that an increase in SNA should theoretically elevate TPR. This finding may either be due to subtle changes in TPR or reduced reliability of the device in measuring TPR under changing HR conditions 58,59. To confirm our hypothesis, future studies should consider directly measuring SNA. In addition, notable differences were found between the two study populations. Our study involved younger participants (average age: 55 years versus 78 years) from a Malaysian population, while the previous study focused on an Australian cohort. Health statuses also differed: our study's participants primarily had pacemakers for sick sinus syndrome, while the previous study involved patients with a heart block 22. Differences in medication use across studies may also have contributed to the observed variations in HR's effects on PPGF.

In the current study, PPGF did not change with increased BP, raising questions about its validity in distinguishing hypertensive from normotensive conditions. Hypertension is a leading risk factor for CVD, and it is associated with structural changes, such as medial thickening and a reduced elastin-to-collagen ratio, resulting in stiffer arteries. If PPGF remains unaffected by BP changes, then this condition may limit its utility in differentiating hypertensive status. To address this issue, the most effective approach is to compare PPGF between normotensive and hypertensive subjects. Previous studies have explored this strategy and found that PPGF is reduced among hypertensive subjects 14. By contrast, our current study examines PPGF response to HR changes within the same individual, where vascular structure remains consistent. This approach allows us to focus specifically on how acute increases in HR and accompanying BP fluctuations influence local blood flow regulation, rather than assessing the effects of chronically elevated BP on the arteriolar structure.

No significant difference between vascular age and chronological age was found in the current study. Given the participants' cardiovascular risk factors, an elevated vascular age was anticipated. However, we hypothesized that the administration of blood flow-enhancing medications contributed to improved PPGF and vascular age. Furthermore, vascular age remained stable across increasing HR. These findings suggest that PPGF and vascular age can serve as reliable vascular markers for assessing CVD risk, even in the presence of varying HRs, particularly among older individuals with multiple comorbidities.

### Study Limitations

The current study has several limitations. (i) This study was organized in subjects with implanted pacemakers and multiple medical problems; thus, most of the participants involved were middle-aged and elderly individuals with cardiac arrhythmias. (ii) We included pacemaker subjects with various indications in this study due to the limited number of subjects with an implanted pacemaker. (iii) We recruited subjects with various types of pacemakers and pharmacological treatments due to the limited number of potential subjects who met the inclusion criteria and under stable condition. This study was conducted based on a small group of highly specific subjects, and thus, its findings could not be directly applied to younger and healthier populations. Lastly, no placebo group was involved. However, our previous study showed that measuring PPG four times at rest, 30 min apart, did not produce any significant differences 15.

## Conclusion

HR variation did not affect PPGF. Therefore, PPGF can be used as a vascular health and vascular age screening tool for the community, particularly among older subjects with multiple medical problems. Future studies should be conducted to expand the findings toward young and healthy populations.

## Figures and Tables

**Table 1:** Inclusion and exclusion criteria

Inclusion Criteria	
1	Patients with pacemaker
2	Age: 18-70 years old
3	Paced rhythm: ≤70 bpm
Exclusion Criteria	
1	Myocardial infarction within the last 12 months of the study
2	Implantable cardioverter defibrillators with pacing function
3	Uncontrolled hypertension (SBP/DBP >160/100 mmHg)
4	LVEF <40%
5	Uncontrolled diabetes mellitus (HbA1c >6%)
6	Uncontrolled congestive heart failure
7	Unstable or stable angina

SBP, systolic blood pressure; DBP, diastolic blood pressure; LVEF, left ventricular ejection fraction; HbA1c, hemoglobin A1c.

**Table 2:** Demographic and biochemical characteristics of the subjects

	N	Range	Mean ± SD
Age (year)	22	29-64	52.27±10.43
Hb (g/L)	22	98-173	135.36±19.05
Platelet (×10^×9^/L)	22	174-394	269.73±57.96
FBS (mmol/L)	22	3.9-14.0	5.71±2.27
TC (mmol/L)	22	3.0-8.0	5.08±1.42
TG (mmol/L)	22	0.46-2.46	1.33±0.51
HDL (mmol/L)	22	0.88-2.20	1.32±0.34
LDL (mmol/L)	22	1.27-5.95	3.15±1.22
UA (mmol/L)	22	0.20-0.57	0.35±0.09
CRP (mg/L)	22	0.1-12.1	3.48±3.37
BMI (kg/m^2^)	22	17.9-30.9	25.96±3.77
SBP baseline (mmHg)	22	114-155	132.59±11.33
DBP baseline (mmHg)	22	55-97	79.82±9.00
PP baseline (mmHg)	22	29-72	52.77±10.59
MAP baseline (mmHg)	22	77.67-115.33	97.41±8.47
HR baseline (bpm)	22	52-82	64.05±7.12
SBP aortic baseline (mmHg)	22	104-174	139.85±18.68
DBP aortic baseline (mmHg)	22	62-105	85.50±12.51
PP aortic baseline (mmHg)	22	37-75	54.34±10.41
AI baseline (%)	22	10.4-62.7	41.31±15.32
PWV baseline (m/s)	22	6.1-13.1	9.46±1.99
PPGF baseline (%)	22	21.97-72.78	51.88±12.44
Vascular age baseline (year)	22	37-94	55.00±13.82

SD, standard deviation; Hb, hemoglobin; FBS, fasting blood sugar; TC, total cholesterol; TG, triglyceride; HDL, high-density lipoprotein; LDL, low-density lipoprotein; UA, uric acid; CRP, C-reactive protein; BMI, body mass index; SBP, systolic blood pressure; DBP, diastolic blood pressure; PP, pulse pressure; MAP, mean arterial pressure; HR, heart rate; AI, augmentation index; PWV, pulse wave velocity; PPGF, finger photoplethysmography fitness index.

**Table 3:** Pacemaker and medication details

No.	Parameter	N
1.	Types of pacemaker	
	Dual-chamber pacemaker	21
	Ventricular sensing and pacing pacemaker	1
2.	Pacing modalities	
	Atrioventricular pacing	21
	Ventricular pacing	1
3.	Implant indications	
	Bradycardia	3
	Sick sinus syndrome	10
	Heart block	8
	Irregular HR	1
4.	Ejection fraction	
	>60%	15
	Data not available	7
5.	Types of medication	
	Angiotensin-converting enzyme inhibitor	6
	Angiotensin receptor blocker	2
	Antiarrhythmic agent	4
	Anticoagulant	3
	Antiplatelet therapy	2
	Acetylsalicylic acid (aspirin)	6
	B-blocker	8
	Calcium channel blocker	7
	Trimetazidine (Vastarel)	3
	Nitroglycerin	2
	Diuretic	2
	Glucophage	3
	Gliclazide	1
	Statin	13
	Ezetimibe	6

N, total number of patients; B, beta.

**Table 4:** Cardiovascular changes due to pacing activity

Parameter	HR 70	HR 80	HR 90	*p*-value
bSBP (mmHg)	128.59±12.98	131.27±12.53	134.32±13.32	0.001
bDBP (mmHg)	79.64±10.64	84.32±10.42	85.86±11.01	<0.001
bMAP (mmHg)	95.95±10.34	99.97±10.21	102.02±10.84	<0.001
PPGF (%)	51.02±11.52	51.15±11.82	49.73±11.55	0.491
Vascular age (year)	55.68±11.54	55.32±11.30	56.59±11.13	0.645

HR, heart rate; *p*-value is a statistical measurement used to validate a hypothesis against observed data; bSBP, brachial systolic blood pressure; bDBP, brachial diastolic blood pressure; bMAP, brachial mean arterial pressure; PPGF, finger photoplethysmography fitness index.

## Abbreviations

IHD: Ischemic heart disease
UA: Uric acid
PPGF: Finger photoplethysmography fitness index
BP: Blood pressure
TG: Triglyceride
CVD: Cardiovascular disease
LDL: Low-density lipoprotein
TC: Total cholesterol
PPG: Finger photoplethysmography
AC: Alternating current
HR: Heart rate
SBP: Systolic blood pressure
AI: Augmentation index
PP: Pulse pressure
DBP: Diastolic blood pressure
CO: Cardiac output
Hb: Hemoglobin
MAP: Mean arterial pressure
AS: Arterial stiffness
AP: Arterial pressure
HIF-1α: Hypoxia-inducible factor-1α
FBS: Fasting blood sugar
SNA: Sympathetic nerve activity
CRP: C-reactive protein
PWV: Pulse wave velocity
HDL: High-density lipoprotein
ECG: Electrocardiogram
BMI: Body mass index
